# Resource theory of quantum scrambling

**DOI:** 10.1073/pnas.2217031120

**Published:** 2023-04-18

**Authors:** Roy J. Garcia, Kaifeng Bu, Arthur Jaffe

**Affiliations:** ^a^Department of Physics, Harvard University, Cambridge, MA 02138

**Keywords:** quantum scrambling, resource theory, magic, quantum entanglement

## Abstract

Quantum chaos has become a cornerstone of physics through its many applications. One trademark of quantum chaotic systems is the spread of local quantum information, which physicists call scrambling. In this work, we introduce a mathematical definition of scrambling and a resource theory to measure it. We also describe two applications of this theory. First, we use our resource theory to provide a bound on magic, a potential source of quantum computational advantage, which can be efficiently measured in experiment. Second, we also show that scrambling resources bound the success of Yoshida’s black hole decoding protocol.

## Significance

The study of chaos has had a significant impact on many scientific disciplines, including quantum physics. One of the hallmarks of quantum chaos is the generation of quantum scrambling, which characterizes information spreading. Here, we introduce a formal definition of scrambling and develop a resource theory to measure it. We validate the usefulness of our resource theory by giving two examples of applications in quantum information, one theoretical and one experimental. Our framework of resource theory provides an approach to study quantum chaos and its applications.

Chaos is a broad area of mathematics with diverse applications to economics, biology, environmental sciences, and physics. In chaotic systems, small perturbations of initial conditions can cause drastic changes to a system’s long-term dynamics; this is known as the butterfly effect (1). Quantum chaos has become central to the study of quantum dynamics by bridging topics such as quantum many-body physics, random matrix theory, and thermalization (2–5). A hallmark of quantum chaotic dynamics is the generation of quantum scrambling, in which local information spreads into the system’s many degrees of freedom. Early studies of information spreading were performed by Lieb and Robinson, in which they established a bound on the speed of information propagation (6). Although the scrambling of quantum chaotic systems has been extensively studied, we provide a rigorous framework to define and measure scrambling.

Scrambling has flourished by connecting diverse areas, including quantum many-body physics (3–5), black hole physics (7–12), and quantum information (7). It has become a prevalent ingredient in many information processing problems found in quantum machine learning (13–17), shadow tomography with classical shadows (18–23), quantum error correction (24, 25), encryption (26), and emergent quantum state designs (27). For instance, scrambling dynamics is used in ref. 27 to generate an ensemble of quantum states that is indistinguishable from the set of all uniformly random states.

To quantify scrambling in a quantum system, several measures have been proposed, such as the average Pauli weight (28–30), the out-of-time-ordered correlator (OTOC) (30–39), the operator entanglement entropy (40–42), and the tripartite mutual information (35, 43). For example, Pan et al. measured the tripartite mutual information as a signature of scrambling on a superconducting quantum processor (44). The OTOC in particular has been used to characterize many-body localization (5, 45–50) and fast scramblers, including black holes and the SYK model (3, 4, 8, 11, 51). Moreover, two mechanisms of scrambling were investigated by measuring the average OTOC and OTOC fluctuations using Google’s Sycamore quantum processor (52).

Exploiting advantages provided by quantum phenomena is one of the key problems in information processing tasks. In recent years, one theoretical framework, called quantum resource theory (53, 54), has been developed to quantify these advantages. In a quantum resource theory, one identifies a set of free quantum states (channels) which do not possess a given resource. All other quantum states (channels) are considered resourceful and are often useful in accomplishing a particular task. A resource monotone quantifies the amount of resource in a state (channel). Using this formalism, it was shown that many features of quantum resources are very general and can be characterized in a unified manner (54, 55). Examples of resources include entanglement (56, 57), magic (58–60), quantum thermodynamics (61–67), coherence (68–75), uncomplexity (76), and quantum heat engines (77, 78), among others.

Resource theories have been widely used to quantify advantages in operational tasks (79). For instance, quantum entanglement is an essential resource for quantum teleportation (80). Magic, which characterizes how far away a quantum state (gate) is from the set of stabilizer states (Clifford gates), has been used in quantum computation to establish bounds on classical simulation times (59, 81–86). Despite its many applications, a resource theory of scrambling has been lacking. Recently, Yoshida conjectured that one may exist (87).

In this work, we introduce a resource theory of scrambling which characterizes two mechanisms, entanglement scrambling and magic scrambling. In entanglement scrambling, resourceful unitaries increase the support of a local Pauli operator. In magic scrambling, resourceful unitaries map a Pauli operator to a sum of multiple Pauli operators. We define resource monotones, Pauli growth and the OTOC magic, to measure each mechanism, respectively. We show that OTOC fluctuations bound the OTOC magic, which provides a theoretical proof of Google’s experimental results (52). We also use our resource theory to bound the decoding fidelity in Yoshida’s decoding protocol (88) for the Hayden–Preskill thought experiment (7), in which scrambling is used to recover a quantum state thrown into a black hole.

## 1. Main Results

### A. Preliminaries.

Given an *n*-qudit system, the generalized *n*-qudit Pauli group is defined as $P_{d}^{⊗n}={\left\{P_{\vec{a}}:P_{\vec{a}}={⊗}_{i=1}^{n}P_{a_{i}}\right\}}_{\vec{a}\in V_{d}^{n}}$, where *d* is the local dimension, $P_{ai}=X^{si}Z^{ti},a_{i}=\left(s_{i},t_{i}\right)\in \upsilon_{d}={\mathbb{Z}}_{d}⊗{\mathbb{Z}}_{d}$ , and $\vec{a}=\left(a_{1},\ldots ,a_{n}\right)$. The generalized Pauli *X* operator is defined by *X*|*j*⟩=|*j* + 1 (mod *d*)⟩, and the generalized Pauli *Z* operator is defined by $Z|j\rangle =e^{2ij\pi /d}|j\rangle$. The inner product between the two *n*-qudit operators *O*_1_ and *O*_2_ is defined as $\left\langle O_{1},O_{2}\right\rangle \equiv \frac{1}{d^{n}}\;\text{Tr}\left\{O_{1}^{†}O_{2}\right\}$. We define the induced norm as ${\left\|\cdot \right\|}_{2}=\sqrt{\left\langle \cdot ,\cdot \right\rangle }$.

Let *O* be an *n*-qudit operator with a norm of ∥*O*∥_2_ = 1. The operator *O* can be expanded in the Pauli basis, $O=\sum_{\vec{a}\in V_{d}^{n}}c_{\vec{a}}P_{\vec{a}}$, where the expansion coefficients satisfy $\sum_{\vec{a}\in V_{d}^{n}}{\left|c_{\vec{a}}\right|}^{2}=1$. Due to this normalization condition, we define $P_{O}\left[\vec{a}\right]\equiv {\left|c_{\vec{a}}\right|}^{2}=\frac{1}{d^{2n}}{\left|\;\text{Tr}\left\{OP_{\vec{a}}\right\}\right|}^{2}$, which implies a probability distribution over $P_{d}^{⊗n}$. The average Pauli weight of *O*, also called the influence (89), is

[1] $$\begin{array}{c} W(O)=\sum_{\vec{a}\in V_{d}^{n}}\left|\vec{a}\right|P_{O}[\vec{a}], \end{array}$$

where $\left|\vec{a}\right|$, the number of *a*_*j*_ in $\vec{a}$ such that *a*_*j*_ ≠ (0, 0), is the Pauli weight of the Pauli operator $P_{\vec{a}}$ (e.g., *I* ⊗ *X* ⊗ *I* ⊗ *Z* has a Pauli weight of 2).

In the following sections, we characterize two mechanisms which we use to construct a definition of scrambling, given below.

Definition 1 An *n*-qudit unitary *U* is a scrambler if it is resourceful in entanglement scrambling, defined in Section B, or magic scrambling, defined in Section C. A unitary is a complete scrambler if it is resourceful in both mechanisms.

### B. Entanglement Scrambling.

We first introduce the framework for entanglement scrambling, in which free unitaries (referred to as nonentangling unitaries) are defined as the unitaries which map any weight-1 Pauli operator by conjugation to an operator with an average Pauli weight of 1. Nonentangling unitaries are generated by swap gates and single-qudit unitaries, as shown in ref. 90. The name of this mechanism is motivated by the fact that nonentangling unitaries do not increase the average of an entanglement measure over all bipartitions^*^.

We define a resource monotone called Pauli growth to measure the generation of entanglement scrambling.

Definition 2 Pauli growth of a unitary *U* is

[2] $$\begin{array}{c} G(U)\equiv \underset{\begin{array}{c} O:\|O{\|}_{2}=1,W(O)=1,\\ \;\text{Tr}\left\{O\right\}=0 \end{array}}{\max}\left[W(U^{†}OU)-1\right]. \end{array}$$

It is proved in ref. 91 that Pauli growth satisfies the following properties, implying that it is a resource monotone,

1. (Faithfulness) *G*(*V*)≥0 for any unitary *V*, and *G*(*U*)=0 iff *U* is a nonentangling unitary,
2. (Invariance) *G*(*U*_1_*VU*_2_)=*G*(*V*) for any unitary *V* and nonentangling unitaries *U*_1_ and *U*_2_.

**Fig. 1.**
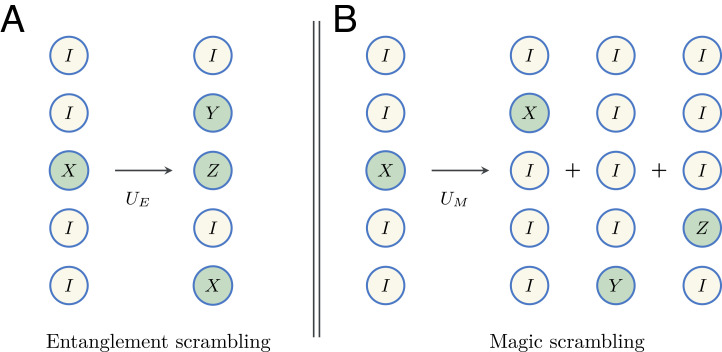
Schematic examples of the two scrambling mechanisms. (*A*) Entanglement scrambling: a unitary *U*_*E*_ maps a weight-1 Pauli operator to a weight-3 Pauli operator via conjugation, i.e., *U*_*E*_^†^(*I* ⊗ *I* ⊗ *X* ⊗ *I* ⊗ *I*)*U*_*E*_ = *I* ⊗ *Y* ⊗ *Z* ⊗ *I* ⊗ *X*. (*B*) Magic scrambling: a unitary *U*_*M*_ maps a weight-1 Pauli operator to a sum of three weight-1 Pauli operators. Here, *U*_*E*_ (*U*_*M*_) generates no magic (entanglement) scrambling.

Faithfulness guarantees that only resourceful unitaries, i.e., unitaries which are not nonentangling, generate entanglement scrambling, indicated by positive Pauli growth. Pauli growth measures the increase in the average Pauli weight of a weight-1 operator under unitary evolution (Fig. 1). In other words, it quantifies operator spreading.

Scrambling in an *n*-qubit system is commonly studied by utilizing the out-of-time-ordered correlator, defined as

[3] $$\begin{array}{c} \;\text{OTOC}\left(U\right)=\left\langle U^{†}P_{D}UP_{A}U^{†}P_{D}UP_{A}\right\rangle , \end{array}$$

where *P*_*A*_ and *P*_*D*_ are Pauli operators which act nontrivially only on the subsystems *A* and *D*, respectively. We define the notation $\left\langle \cdot \right\rangle \equiv \frac{1}{2^{n}}\;\text{Tr}\left\{\cdot \right\}$. The OTOC is related to commutator growth via ∥[*U*^†^*P*_*D*_*U*, *P*_*A*_]∥_HS_^2^ = 2^*n* + 1^(1−OTOC(*U*)), where ∥ ⋅ ∥_HS_ denotes the Hilbert–Schmidt norm. For disjoint subsystems *A* and *D*, this commutator norm measures the spread of the support of *P*_*D*_ to the subsystem *A* after conjugation by *U*. A small OTOC value is traditionally considered a signature of scrambling. In the large *n* limit, the OTOC of *U* and Pauli growth satisfy the following relation (see ref. 91 for a proof, which is based on the results in ref. 90):

[4] $$\begin{array}{c} \begin{array}{c} \underset{A}{E}\underset{P_{A}\neq I_{A}}{E}\;\text{OTOC}\left(U\right)\geq 1-\frac{4}{3n}\left(G(U)+1\right), \end{array} \end{array}$$

where *D* is the *n*-th qubit, *A* is any other single-qubit subsystem, $\underset{A}{E}$ is the uniform average over all choices of *A*, and $\underset{P_{A}\neq I_{A}}{E}$ is the uniform average over all nonidentity Pauli operators on *A*. The OTOC is hence an indicator of operator spreading.

### C. Magic Scrambling.

We now introduce the framework for magic scrambling. A free unitary is defined to map any Pauli operator to a Pauli operator (up to a phase) under conjugation. By definition, free unitaries are Clifford unitaries. Resourceful unitaries are non-Clifford unitaries; they can map a Pauli operator to a superposition of Pauli operators, i.e., they generate magic scrambling (Fig. 1). Magic monotones quantify the distance between a unitary and the set of Clifford unitaries. This framework is identical to the resource theory of magic, but we refer to it as magic scrambling to emphasize its operational interpretation.

We introduce a magic monotone, which we call the OTOC magic.

Definition 3 The OTOC magic of an *n*-qubit unitary *U* is

[5] $$\begin{array}{c} O_{M}(U)\equiv \underset{P_{\vec{a}},P_{\vec{b}}\in P_{2}^{⊗n}}{\max}\left[1-\left|\text{OTOC}(U)\right|\right], \end{array}$$

where $P_{2}$ is the qubit Pauli group and OTOC(*U*)= $\left\langle U^{†}P_{\vec{a}}UP_{\vec{b}}U^{†}P_{\vec{a}}UP_{\vec{b}}\right\rangle$.

In ref. 91, we prove that the OTOC magic satisfies the following monotone properties:

1. (Faithfulness) *O*_*M*_(*V*)≥0 for any unitary *V*, and *O*_*M*_(*U*)=0 iff *U* is a Clifford unitary,
2. (Invariance) *O*_*M*_(*U*_1_*VU*_2_)=*O*_*M*_(*V*) for any unitary *V* and Clifford unitaries *U*_1_ and *U*_2_.

We compute the OTOC magic for two examples of gates in the Clifford hierarchy (92). The *k*-th level of the Clifford hierarchy is defined as $C^{\left(k\right)}=\left\{U\in U\left(n\right):U^{†}P_{2}^{⊗n}U\subset C^{\left(k-1\right)}\right\}$, where $C^{\left(1\right)}$ is the Pauli group and $C^{\left(2\right)}$ is the Clifford group.

Example 1 All non-Clifford unitaries in the third level of the Clifford hierarchy maximize the OTOC magic (see ref. 91 for a proof):

[6] $$\begin{array}{c} O_{M}\left(U\right)=1,\;\;\;\forall U\in C^{\left(3\right)}\backslash C^{\left(2\right)}. \end{array}$$

Example 2 Define the single-qubit phase shift gate as

[7] $$\begin{array}{c} U_{\varepsilon }=\left(\begin{array}{cc} 1 & 0\\ 0 & e^{i\varepsilon } \end{array}\right), \end{array}$$

where *ε* ∈ [0, 2*π*). Its OTOC magic is *O*_*M*_(*U*_*ε*_)= 1 − |cos(2*ε*)| (see ref. 91 for a proof). Let $\varepsilon_{k}=\frac{\pi }{2^{k-1}}$ for any integer *k* >  0. Then, $U_{\varepsilon k}\in C^{\left(k\right)}$ (93) and the OTOC magic is

[8] $$\begin{array}{c} O_{M}\left(U_{\varepsilon_{k}}\right)=1-\left|\cos\left(\frac{\pi }{2^{k-2}}\right)\right|. \end{array}$$

**Fig. 2.**
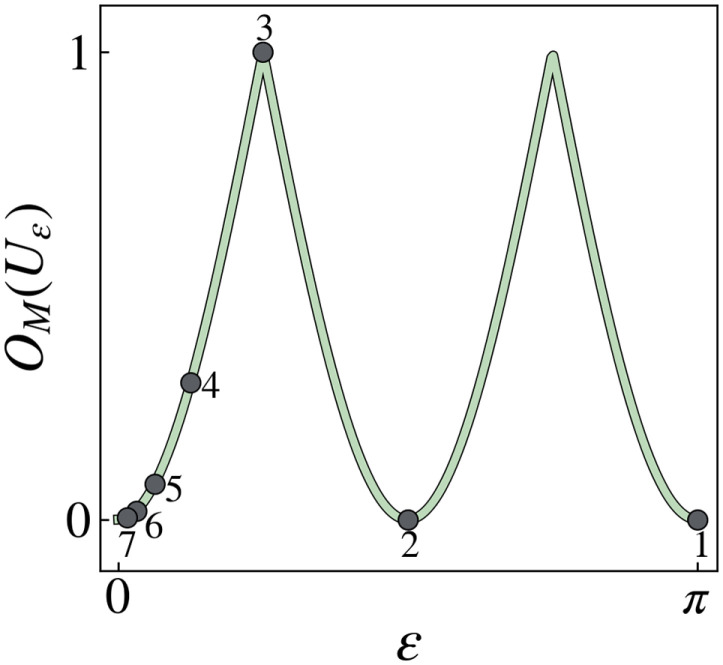
Plot of *O*_*M*_(*U*_*ε*_) with respect to *ε*. Numbered points label the values of *O*_*M*_(*U*_*ε*_*k*__) when $\varepsilon_{k}=\frac{\pi }{2^{k-1}}$ and *k* = 1, 2, …, 7. The *k*-th point corresponds to the OTOC magic of a unitary in the *k*-th level of the Clifford hierarchy. For *k* ≥ 3, the OTOC magic decreases with increasing *k*.

Since gates in the *k*-th level of the Clifford hierarchy map Pauli operators to gates in the *k* − 1 level, one may be tempted to interpret the level of the hierarchy as a measure of a gate’s “distance” from the second level (i.e., as a measure of magic). However, according to (**8**), for *k* ≥ 3, the OTOC magic of *U*_*ε*_*k*__ decreases as *k* increases, indicating that some gates in the higher levels of the Clifford hierarchy can have a small amount of magic (Fig. 2).

The OTOC magic can be measured via OTOC measurement protocols based on a randomized measurement toolbox (94, 95), classical shadows (22), and teleportation (96). Such protocols allow one to measure the OTOC by circumventing measurement techniques such as time reversal (97–99). Other magic monotones (100–102) have also been theoretically related to OTOCs.

We develop a scrambling resource theory based on two separate mechanisms, as some unitaries may be free with respect to one mechanism, but resourceful with respect to the other. For example, the T gate is a free entanglement scrambling unitary, but it is resourceful in magic scrambling. The CNOT gate is a Clifford unitary, but it is resourceful in entanglement scrambling. In the case where a unitary is resourceful in both mechanisms, it can map a weight-1 Pauli operator to a superposition of multiple high-weight Pauli operators. This unitary mapping is consistent with the traditional qualitative description of scrambling (103).

### D. Application to Google’s Experimental Results.

Quantum supremacy, the realization of a quantum computational speed-up, has been claimed in random circuit sampling problems (104, 105). Magic has been suggested as a potential source of quantum computational advantage due to the Gottesman-Knill theorem (106). Computing magic monotones often requires an exponential number of measurements (e.g., the OTOC magic necessitates a maximization over an exponentially large set). Constructing bounds on magic which can be measured efficiently permits the quantification of this resource in large-scale systems. We construct such a bound in this section.

The OTOC fluctuations^†^, defined as

[9] $$\begin{array}{c} \delta =\sqrt{\underset{U∼E}{E}{\left|\;\text{OTOC}\left(U\right)-\underset{V∼E}{E}\;\text{OTOC}\left(V\right)\right|}^{2}}, \end{array}$$

where the averages are taken over a random unitary ensemble ℰ, have been measured by Google on the Sycamore quantum processor (52). Small fluctuations were shown to be evidence of magic and operator entanglement. When ℰ is the Clifford ensemble, $\underset{U∼E}{E}\;\text{OTOC}\left(U\right)\to 0$ for large systems and OTOC(*U*) fluctuates between +1 and −1, implying that *δ* = 1. However, when *U* is sampled from a non-Clifford ensemble, then |OTOC(*U*)| ≤ 1. It was shown in this experiment that OTOC fluctuations decay as the magic of the unitaries in the ensemble is increased. Also, it was numerically shown in ref. 107 that the average mana, a magic monotone, increases as the OTOC fluctuations decrease. Here, we establish an inequality between *δ* and the OTOC magic.

Theorem 1 If $\underset{U∼E}{E}\;\text{OTOC}\left(U\right)\to 0$, then

[10] $$\begin{array}{c} \underset{U∼E}{E}O_{M}\left(U\right)\geq 1-\delta . \end{array}$$

We prove Theorem 1in ref. 91. Measuring OTOC fluctuations near 0 indicates that, on average, the OTOC magic is near maximal. This provides a resource-theoretic interpretation of the small OTOC fluctuations measured in Google’s experiment^‡^.

We provide numerical simulations to support the bound in (**10**). The ensemble ℰ consists of 4-qubit unitaries of the form:
[11]
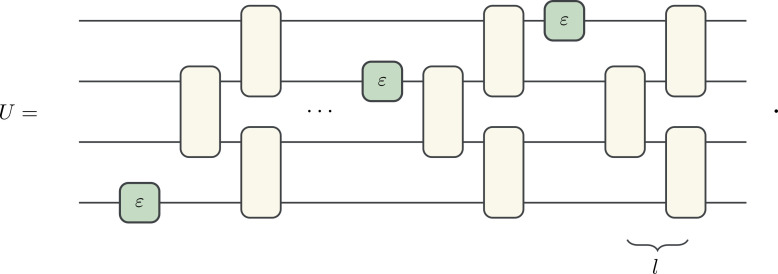


**Fig. 3.**
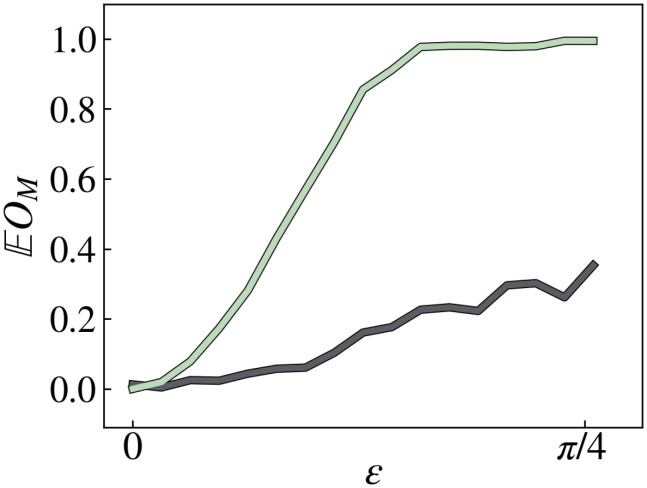
Plot of $\underset{U∼E}{E}O_{M}\left(U\right)$ (green) and 1 − *δ* (black) against *ε*. These quantities are empirically computed using 50 random samples from the ensemble ℰ generated by Eq. **11**.

The 2-qubit unitaries are randomly sampled from the Clifford group and are arranged in a brick-work architecture with *l* = 4 layers. The four single-qubit unitaries are each *U*_*ε*_, defined in Eq. **7**, with a fixed value of *ε*. We compute the fluctuations of the OTOC, ⟨*U*^†^*X*_1_*UZ*_4_*U*^†^*X*_1_*UZ*_4_⟩, where *X*_1_ denotes a Pauli *X* operator on the first qubit. The OTOC magic of *U* is tuned by varying *ε*. Fig. 3 plots $\underset{U∼E}{E}O_{M}\left(U\right)$ and 1 − *δ* against *ε*. Both are positively correlated with *ε*, and $\underset{U∼E}{E}O_{M}\left(U\right)$ is bounded by 1 − *δ* from below.

Although the bound in Theorem 1is not optimal for the particular unitary considered in Eq. **11**, the bound is better for generic unitaries. In the case that *U* is randomly sampled 50 times from the Haar measure on the 4-qubit unitary group, we numerically computed that $\underset{U∼E}{E}O_{M}\left(U\right)=0.999998$ and 1 − *δ* = 0.911131. We believe that Theorem 1will be practical for bounding the magic of deep random quantum circuits in experiments.

### E. Application to Hayden–Preskill Decoding Protocol.

We apply the resource theory of scrambling to the Hayden–Preskill thought experiment (7), an information recovery problem. In this thought experiment, a quantum state is thrown into an *n*-qubit black hole. The black hole’s scrambling dynamics, *U*_bh_, lead to delocalization of the state’s information. By collecting the emitted Hawking radiation, one can decode the state thrown in.

**Fig. 4.**
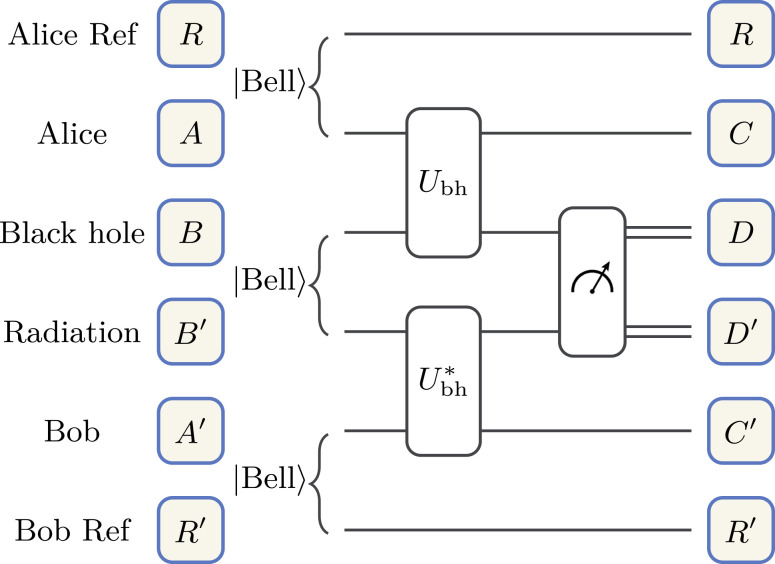
Quantum circuit for the Hayden–Preskill decoding protocol. Alice maximally entangles her state, defined on the system *A*, with a reference system *R*. Bob maximally entangles his system *A*′ with a reference system *R*′. Alice throws her state into the black hole, system *B*. Afterward, the new black hole, system *AB*, evolves under the unitary *U*_bh_. Bob applies the unitary *U*_bh_^*^ to his system *B*′*A*′, where *B*′ represents the Hawking radiation emitted by the black hole before Alice threw her state in. Bob then projectively measures a Bell state, $|{\mathrm{Bell}\rangle }_{D,D\prime }=\frac{1}{\sqrt{2^{n}}}\sum_{i=1}^{2^{n}}{\left|i\right\rangle }_{D}⊗{\left|i\right\rangle }_{D\prime }$, between systems *D* and *D*′. System *D* represents the Hawking radiation emitted after Alice throws her state in. If successful decoding occurs, then Bob’s reference system *R*′ forms a Bell state with *R*.

Yoshida et al. (87, 88, 108) constructed a teleportation-based decoding protocol to recover the input state thrown into the black hole with a decoding fidelity of *F*(*U*_bh_). See Fig. 4 for a description. The decoding fidelity is determined by the average OTOC:

[12] $$\begin{array}{c} \underset{P_{A},P_{D}}{E}\;\text{OTOC}\left(U_{\;\text{bh}}\right)=\frac{1}{d_{A}^{2}F\left(U_{\;\text{bh}}\right)}, \end{array}$$

where OTOC(*U*_bh_)=⟨*U*_bh_^†^*P*_*D*_*U*_bh_*P*_*A*_*U*_bh_^†^*P*_*D*_*U*_bh_*P*_*A*_⟩ and *d*_*A*_ = 2^*n*_*A*_^ is the Hilbert space dimension of system *A*. The average is taken uniformly over all Pauli operators on the systems *A* and *D*, defined in Fig. 4.

We establish an inequality relating the decoding fidelity and the Pauli growth of *U*_bh_ (see ref. 91 for a proof ^§^).

Theorem 2 Let *D* be the *n*-th qubit, and let *A* be any other single-qubit system. Let $\underset{A}{E}$ denote the uniform average over all such *A* systems. In the large *n* limit, the decoding fidelity *F*(*U*_bh_) and the Pauli growth of *U*_bh_ satisfy the following inequality:

[13] $$\begin{array}{c} \begin{array}{c} \underset{A}{E}\frac{1}{F(U_{\;\text{bh}})}\geq \left(d_{A}^{2}-1\right)\left[1-\frac{4}{3n}\left(G(U_{\;\text{bh}})+1\right)\right]+1. \end{array} \end{array}$$

This inequality illustrates the utility of scrambling resources, as measured by Pauli growth, in recovering quantum information.

## 2. Conclusion

We have introduced a resource theory composed of two mechanisms to define and measure quantum scrambling. In the entanglement scrambling mechanism, we introduce Pauli growth as a monotone to measure operator spreading. In the magic scrambling mechanism, we introduce the OTOC magic as a monotone to measure the generation of operator entanglement. We use these monotones to bound the OTOC fluctuations measured in Google’s experiment (52) and to bound the success of Yoshida’s decoding protocol. These applications provide an operational interpretation of our resource monotones.

We propose that these monotones may also be used to bound other scrambling tools, such as the tripartite mutual information, the operator entanglement entropy, and Lyapunov exponents in OTOC dynamics. Furthermore, we conjecture that this scrambling resource theory can be generalized to quantum channels, which may be useful in understanding noise effects.

## Data Availability

There are no data underlying this work.

## Footnotes

^*^For example, the average Rényi-2 entanglement entropy, ${\bar{S}}^{\left(2\right)}\left(\rho \right)\equiv \frac{1}{2^{n}}\sum_{A\in \left[n\right]}-\log\text{Tr}\left\{\rho_{A}^{2}\right\}$ , where ρ*A* is the reduced state of an *n*-qudit state ρ on a subsystem *A*, satisfies ${\bar{S}}^{\left(2\right)}\left(U\rho U^{†}\right)={\bar{S}}^{\left(2\right)}\left(\rho \right)$ , where *U* is a nonentangling unitary (90).

^†^In this experiment, the OTOC is defined as an expectation value with respect to a pure state, but we utilize the maximally mixed state, as in Eq. **3**.

^‡^In fact, a small value of $\left|\text{OTOC}\left(U\right)\right|$ is an indication of the magic of *U*, since it bounds *O_M_(U)*.

^§^More generally in Theorem 2, *D* can be a randomly selected, single-qubit system.
